# Feasibility of aligning creatine kinase MB activity and mass data in multicentre trials using generalized additive modelling

**DOI:** 10.1093/icvts/ivae138

**Published:** 2024-07-23

**Authors:** Markus Hoenicka, Arbresha Vokshi, Shaoxia Zhou, Andreas Liebold, Benjamin Mayer

**Affiliations:** Department of Cardiothoracic and Vascular Surgery, Ulm University Medical Center, Ulm, Germany; Department of Cardiothoracic and Vascular Surgery, Ulm University Medical Center, Ulm, Germany; Department of Clinical Chemistry, Ulm University Medical Center, Ulm, Germany; Department of Cardiothoracic and Vascular Surgery, Ulm University Medical Center, Ulm, Germany; Institute for Epidemiology and Medical Biometry, Ulm University, Ulm, Germany

**Keywords:** Creatine kinase MB, Generalized additive models, Statistical modelling, Myocardial injury

## Abstract

**OBJECTIVES:**

Elevated serum creatine kinase isoenzyme MB (CK-MB) levels indicate myocardial ischaemia and periprocedural myocardial injury during treatment of heart diseases. We established a method to predict CK-MB mass from activity data based on a prospective pilot study in order to simplify multicentre trials.

**METHODS:**

38 elective cardiac surgery patients without acute myocardial ischaemia and terminal renal failure were recruited. CK-MB mass and activity were determined in venous blood samples drawn preoperatively, postoperatively, 6 h post-op, and 12 h post-op. Linear regression and generalized additive models (GAMs) were applied to describe the relationship of mass and activity. Influences of demographic and perioperative factors on the fit of GAMs was evaluated. The agreement of predicted and measured CK-MB masses was assessed by Bland–Altman analyses.

**RESULTS:**

Linear regression provided an acceptable overall fit (*r*^2^ = 0.834) but showed deviances at low CK-MB levels. GAMs did not benefit from the inclusion of age, body mass index and surgical times. The minimal adequate model predicted CK-MB masses from activities, sex and sampling time with an *r*^2^ of 0.981. Bland–Altman analyses confirmed narrow limits of agreement (spread: 8.87 µg/l) and the absence of fixed (*P* = 0.41) and proportional (*P* = 0.21) biases.

**CONCLUSIONS:**

GAM-based modelling of CK-MB data in a representative patient cohort allowed to predict CK-MB masses from activities, sex and sampling time. This approach simplifies the integration of study centres with incompatible CK-MB data into multicentre trials in order to facilitate inclusion of CK-MB levels in statistical models.

## Graphical Abstract

**Figure:**
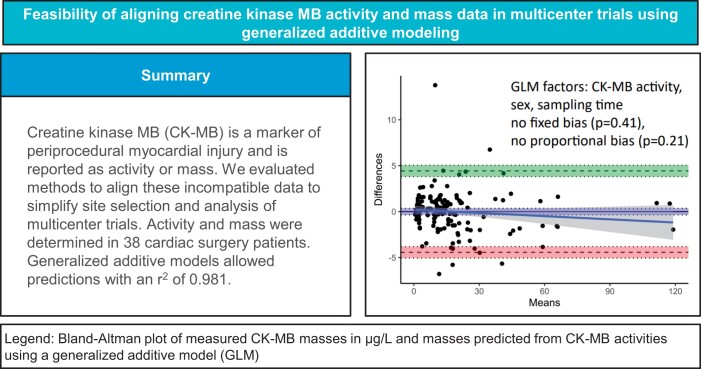

## INTRODUCTION

Creatine kinase (CK; EC 2.7.3.2) is a dimer of M and B subunits. The MB isoenzyme (CK-MB) is most abundant in myocardium [1]. Increased serum levels of CK-MB may indicate myocardial damage, but also skeletal muscle trauma. Although CK-MB was gradually replaced by the earlier detectable and more specific biomarkers cardiac troponin T and I [2], it is considered a robust biomarker for monitoring non-ischaemic periprocedural myocardial injury irrespective of a patient’s kidney function, for detecting early postoperative reinfarction [3], and for trials evaluating myocardial protection in cardiac surgery. Two metrics quantify CK-MB serum levels. CK-MB activity is reported as units per serum volume (U/l), and CK-MB protein concentration (“mass”) is reported as µg/l. CK-MB mass is recommended for the diagnosis of myocardial infarction if no troponin assay is available [4]. The existence of two incompatible measures of CK-MB makes the comparison of studies inconvenient, and places a burden on the design of multicentre studies which plan to evaluate biomarker levels.

Improved CK-MB assays made frequent method comparisons necessary [1, 5, 6]. Lévesque *et al.* [7] observed a “good agreement” between their activity and mass assays, although the Bland–Altman plot [8] showed a substantial proportional bias. The authors did not observe significant differences between sexes and did not evaluate age as a factor. Marwah *et al.* [9] determined CK-MB in patients with acute coronary syndrome (ACS). The correlation between activity and mass assays was best in serum samples obtained 12–24 h after admission, whereas correlation was poorer before and even more so after this time frame. Influences of sex or age were not investigated.

A linear relationship between activity and mass data is a reasonable assumption. Slope and intercept of a linear regression [7] would allow a simple transformation. However, there is evidence that CK-MB serum levels are affected by age [10], sex [11–13], ethnicity [11], sampling time [9] and even geographical factors [14]. Mass and activity assays may be affected differently by these factors. Therefore, more versatile methods, such as the non-parametric generalized additive models (GAM), come in handy to describe the relationship between data from different types of assays [15].

This study was undertaken to optimize the conversion of CK-MB activity to mass data for the prospective multicentre trial “Safety and Effectiveness of Endoaortic Balloon Occlusion as Compared to Transthoracic Clamping for Minimally Invasive Mitral Valve Surgery” (SECRET, trial ID DRKS00027353). CK-MB activity and mass were determined concurrently in cardiac surgery patients of one of the SECRET study centres to generate a training dataset for modelling purposes. We investigated whether non-parametric multivariable statistical modelling with additional factors such as age, sex and time of sampling improves the interconversion results.

## PATIENTS AND METHODS

### Ethical statement

The prospective observational study was approved by the ethics committee of Ulm University (file no. 84/23) and complied with the principles of the Declaration of Helsinki. Written informed consent was obtained from each patient prior to enrolment.

### Patient enrolment

Patients were recruited in the Department of Cardiothoracic and Vascular Surgery, Ulm University Medical Center, Ulm, Germany, in May and June 2023. Patients of at least 18 and not exceeding 80 years of age who were scheduled for elective cardiac surgery with an extracorporeal circulation were eligible. Exclusion criteria were reoperations, acute myocardial infarction, ACS, terminal renal failure, high-dose biotin therapy and inability to provide written informed consent.

### Sample collection

Five mL of venous blood were collected in serum monovettes (Sarstedt, Nümbrecht, Germany) at 4 time points: preoperative, immediately postoperative, 6 h postoperative and 12 h postoperative. Samples were processed as per the collection tube instructions, and the obtained serum was stored in aliquots at −80°C immediately.

Patient data were limited to common and routinely available parameters. Age, sex, body mass index (BMI), underlying disease, comorbidities and risk factors were recorded during anamnesis. Perioperative data included the type and duration of surgery, and the bypass and cross-clamp times.

### CK-MB determination

Serum sample storage times at −80°C did not exceed the recommendations of both CK-MB assays used. Thawed serum samples were batch-processed in the Department of Clinical Chemistry, Ulm University Medical Center. CK-MB activities were determined by an immunological UV assay (CKMB, Roche Diagnostics, Mannheim, Germany) on a Cobas c 503 analyzer (Roche Diagnostics). In case of elevated preoperative CK-MB activities, total CK activity was measured on the same instrument (CK, Roche Diagnostics) as recommended by the CK-MB kit manual. CK-MB mass was determined in the same sample by the electrochemiluminescence immunoassay CK-MB STAT (Roche Diagnostics) on a Cobas e 801 system (Roche Diagnostics).

Comorbidities may induce the formation of high-molecular CK complexes known as macro-CK [16]. Patients with this condition display normal CK-MB masses but elevated CK-MB activities in the absence of myocardial damage [17]. According to the CK-MB activity kit manual, CK-MB activities exceeding 25% of the total CK activity indicate macro-CK. Patients with preoperative CK-MB activities exceeding this limit were excluded from the analysis.

### Data analysis

All statistical analyses were performed with the software R (R Foundation for Statistical Computing, Vienna, Austria) version 4.1.2 and additional R packages as noted. All analyses were pre-specified. All reported p values are two-sided.

### Determination of sample size

The inclusion of 160 serum samples from 40 patients surpasses the sizes of previous method comparison studies [7, 9] and meets the recommendation of the developers of the Bland–Altman method [18].

### Demographic data

Binary parameters were described as frequencies. Continuous numeric parameters were evaluated as mean and standard deviation (SD) for normally distributed data and as median and interquartile range for non-normally distributed data. The influence of sex and sampling time on CK-MB activities or masses were analysed by fitting the linear mixed-effects model CK_MB∼sex*sampling_time, using patients as a source of random errors (nlme package).

### Regression analysis

CK-MB activity was modelled as a function of CK-MB mass using the simple linear model CK_MB_Activity∼CK_MB_Mass. The model was fitted by ordinary least squares (OLS) regression.

### Generalized additive models

CK-MB mass was modelled as a function of CK-MB activity and other demographic and perioperative factors using GAMs (mgcv package). Several models were constructed. The simple model CK_MB_Mass∼s(CK_MB_Activity) was equivalent to the linear regression model except for the use of a smoother (the “s()” term in the model specification) instead of a regression line. Multivariable model exploration started with the maximal model CK_MB_Mass∼s(CK_MB_Activity, by=sex) + s(age, by=sex) + s(BMI, by=sex) + s(surgery_time, by=sex) + s(bypass_time, by=sex) + s(cross_clamp_time, by=sex) + s(sampling_time) + ti(sampling_time, CK_MB_Activity, bs=“re”) + ti(patient, CK_MB_Activity, bs=“re”). The model included 2 random error terms for CK-MB activity (the “ti()” terms). They addressed the longitudinal within-subject variability and the between-subjects variation of CK-MB activities. As the reference ranges of both CK-MB activity and mass differ between sexes [11, 19], the smoothers were stratified by sex. This model was simplified step-wise until a minimum of Akaike's information criterion (AIC) was reached. The coefficient of determination (*r*^2^) and the generalized cross-validation (GCV) score were used to assess the quality of the models, and diagnostic plots were used to assess the distribution of residuals, their dependence on the predictor, and the relationship of measured and fitted data.

### Conversion method comparison

Predicted CK-MB masses were calculated from the measured activities by means of the previously established regression coefficients or by prediction from a GAM. The differences between these predicted masses and the corresponding measured masses were plotted against their means according to the Bland–Altman method [8].

The influence of the sampling time on the differences between predicted and measured masses diff was assessed by fitting the linear mixed-effects model diff∼sampling_time, using random=∼1|patient to specify patients as a source of random errors. Significant differences between the levels of sampling time were identified by pairwise comparisons using Tukey's range test (multcomp package).

## RESULTS

### Patient characteristics

**Figure 1:**
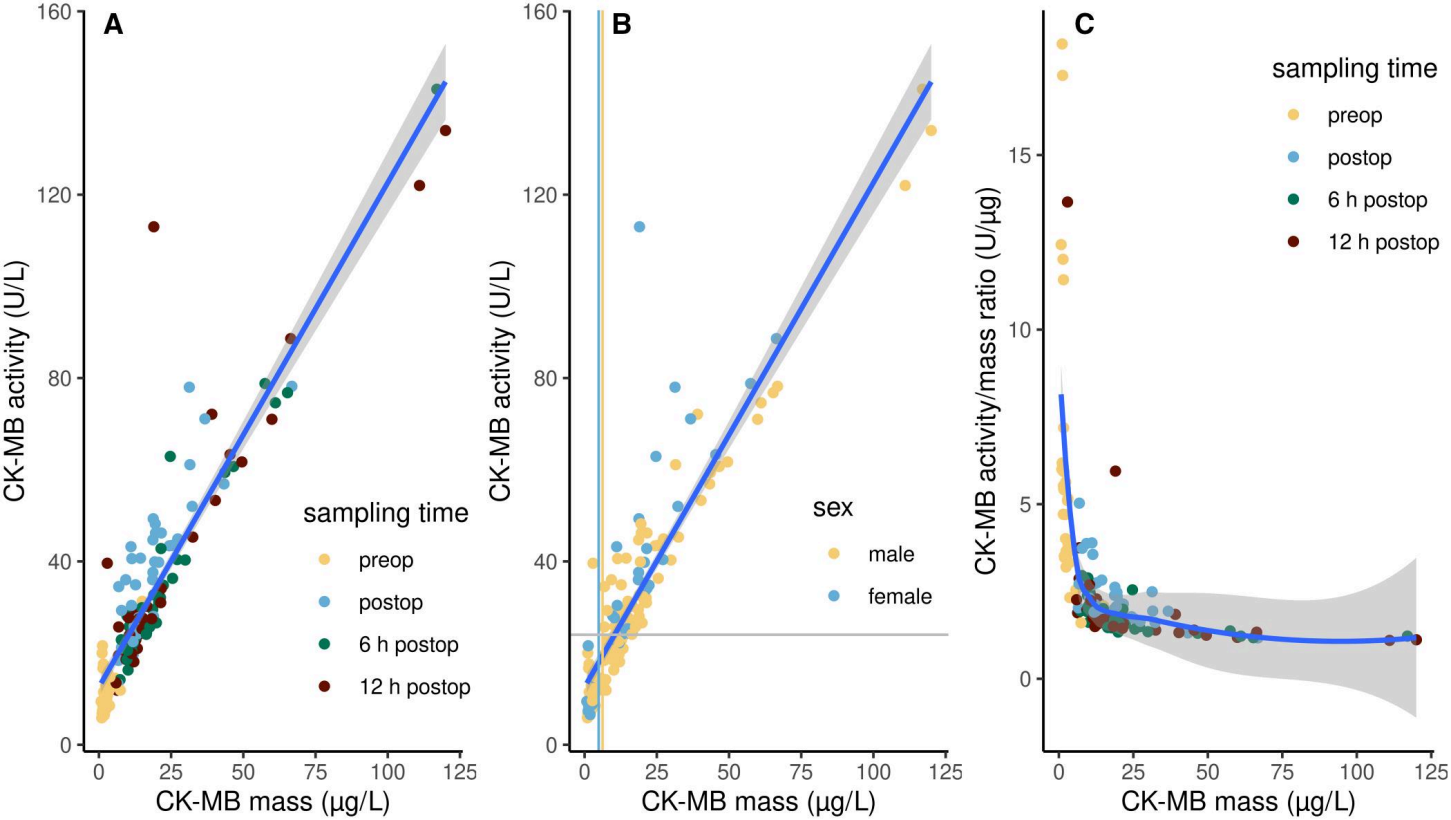
Scatter plots of CK-MB levels measured as activity, mass concentration, and their ratio in 152 serum samples of 38 patients. (**A**) CK-MB activity vs mass. Colour indicates sampling time. (**B**) CK-MB activity vs mass. Colour indicates sex. The straight blue lines in (A) and (B) represent the OLS linear regression with the 95% confidence interval in dark grey. The light grey horizontal line indicates the 99th percentile of CK-MB activity in healthy subjects. The yellow and blue vertical lines indicate the 99th percentiles of CK-MB masses of male and female healthy subjects, respectively. (**C**) CK-MB activity/mass ratios vs mass. Colour indicates sampling time. The blue line is a locally estimated scatterplot smoothing non-parametric regression with the 95% confidence interval displayed in grey.

Two patients were excluded from the analysis owing to macro-CK. Demographic and perioperative data of the remaining 38 study patients are shown in Supplementary Material, Table S1. The leading risk factors were hypertension and hyperlipidaemia in 95% and 84% of the patients, respectively. The most common procedure was coronary artery bypass grafting (58%). More than half of the patients required combined procedures. Two patients had slightly elevated CK-MB mass levels preoperatively (15.0 and 7.4 µg/l). All patients showed elevated CK-MB levels immediately after surgery and at 6 h post-op (Fig. 1) with no clinical signs of ACS. CK-MB masses of 2 patients had returned to below the 99th percentile at 12 h post-op.

### CK-MB activity and mass

The relationship of CK-MB activity and mass appeared to be linear (Fig. 1, Supplementary Material, Model S1). Median CK-MB activities were 25.75 U/l (16.60–39.83 U/l) and ranged from 5.88 to 143.00 U/l. Median CK-MB masses were 12.40 µg/l (6.22–20.00 µg/l) with 0.76 and 120.00 µg/l as the lowest and highest readings, respectively. Female patients had lower basal CK-MB levels for both activity and mass, but slightly higher levels at all postoperative sampling times (Supplementary Material, Table S2). A mixed-effects model analysis did not consider the influence of sex significant (*P* = 0.45 for activity, *P* = 0.92 for mass), but the influence of sampling time was significant for both CK-MB readouts (*P* < 0.001) with no interaction of both factors. The ratio of both measurements depended on sampling time (*P* < 0.001) but not on sex (*P* = 0.43). The ratio of CK-MB activity and mass was highest in preoperative samples and lowest in samples taken 6 h postoperatively. Ratios were exceptionally high in samples with the lowest CK-MB masses (Fig. 1C).

### Comparison of CK-MB data conversion methods

The relationship of CK-MB activity and mass data was described by a linear regression model and GAMs. To assess the utility of these models to convert activity into mass data, CK-MB masses were predicted from the activity data and compared to the measured masses.

**Figure 2:**
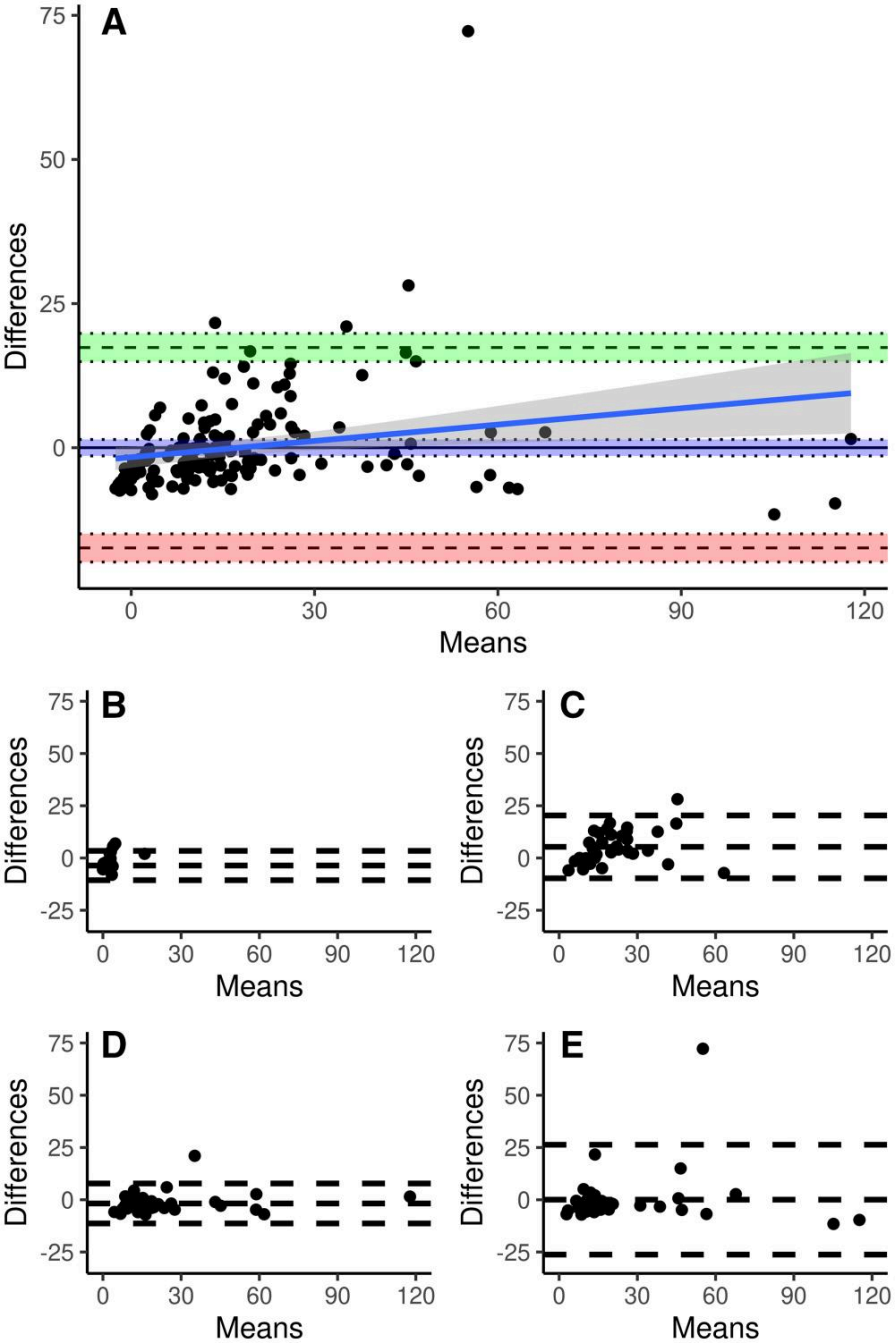
Bland–Altman plots comparing measured CK-MB masses and masses predicted from CK-MB activities with an OLS linear regression. Each panel shows a plot of the differences between measured and predicted masses against their mean. (**A**) All samples (*n* = 152). The areas between the pairs of dotted lines indicate the 95% confidence intervals around the upper limit of agreement (green), the mean difference (blue) and the lower limit of agreement (red). The blue line is the linear regression of differences and means with the 95% confidence interval in dark grey. (**B**) Preoperative samples (*n* = 38). (**C**) Immediately postoperative samples (*n* = 38). (**D**) 6 h postoperative samples (*n* = 38). (E) 12 h postoperative samples (*n* = 38).

OLS regression suggested a conversion according to the formula Mass=(Activity-12.586)/1.100 (Fig. 1) with an adjusted *r*^2^ of 0.834. The corresponding Bland–Altman plot (Fig. 2A) indicates a reasonable agreement of predicted and measured CK-MB masses. There was no fixed bias (*P* = 0.078) but a significant proportional bias (*P* = 0.007) towards higher differences with increasing means. The upper and lower limits of agreement (LoA) were determined as 17.38 µg/l (95% CI: 14.95–19.82 µg/l) and −17.38 µg/l (CI: −19.82 to −14.95 µg/l), respectively, resulting in a spread of data between the LoAs of 34.77 µg/l.

The simple GAM resulted in an adjusted *r*^2^ value of 0.867 (GCV 55.824, AIC 1044.225) (Supplementary Material, Model S2). The maximal model resulted in an adjusted *r*^2^ of 0.979 (GCV 12.728, AIC 798.1032), but it contained non-significant terms (Supplementary Material, Model S3). Step-wise simplification led to the minimal adequate model:

$$\begin{array}{c} \mathrm{CK}\_\mathrm{MB}\_\mathrm{Mass}∼s(\mathrm{CK}\_\mathrm{MB}\_\mathrm{Activity},\mathrm{by}=\mathrm{sex})\\ +s\left(\mathrm{sampling}\_\mathrm{time}\right)+\mathrm{ti}(\mathrm{sampling}\_\mathrm{time},\mathrm{CK}\_\mathrm{MB}\_\mathrm{Akt},\mathrm{bs}=¨\mathrm{re}¨)\\ +\mathrm{ti}(\mathrm{patient},\mathrm{CK}\_\mathrm{MB}\_\mathrm{Activity},\mathrm{bs}=¨\mathrm{re}¨) \end{array}$$

**Figure 3:**
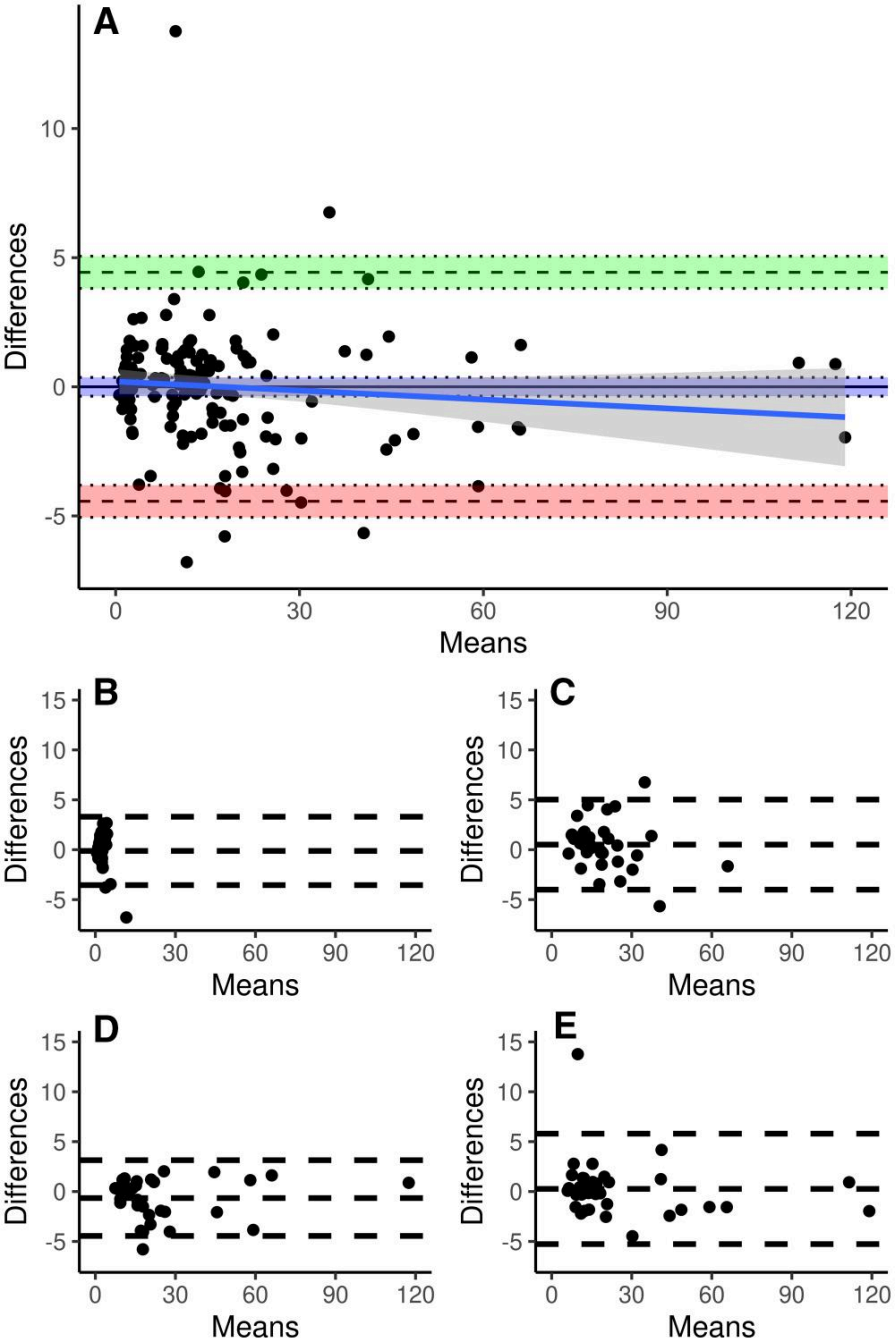
Bland–Altman plots comparing measured CK-MB masses and masses predicted from CK-MB activities with the minimal adequate GAM. Each panel shows a plot of the differences between measured and predicted masses against their mean. Please note the approximately 5-fold narrower range of the differences compared to Fig. 2. (**A**) All samples (*n* = 152). The areas between the pairs of dotted lines indicate the 95% confidence intervals around the upper limit of agreement (green), the mean difference (blue) and the lower limit of agreement (red). The blue line is the linear regression of differences and means with the 95% confidence interval in dark grey. (**B**) Preoperative samples (*n* = 38). (**C**) Immediately postoperative samples (*n* = 38). (**D**) 6 h postoperative samples (*n* = 38). (E) 12 h postoperative samples (*n* = 38).

All terms of this model were considered significant (Supplementary Material, Model S4, adjusted *r*^2^ 0.981, GCV 10.816, AIC 776.079). The Bland–Altman plot (Fig. 3A) suggested a good agreement of predicted and measured masses. There was neither a fixed (*P* = 0.41) nor a proportional (*P* = 0.21) bias. The upper and lower LoAs were 4.43 µg/l (CI: 3.81–5.05 µg/l) and −4.43 µg/l (CI: −5.05 to −3.81 µg/l), respectively. There was a spread of data between LoAs of 8.87 µg/l.

Sampling time affected the differences between measured masses and masses predicted with OLS regression (*P* < 0.001; Supplementary Material, Table S3 and Fig. 2). Values of immediately postoperative samples differed significantly from values of preoperative samples and of 6 and 12 h post-op samples (1 vs 0 and 1 vs 2 *P* < 0.001; 1 vs 3 *P* = 0.002). The Bland–Altman analysis found a fixed bias at the first 3 sampling times and an excessive spread at the last sampling time. The spread varied from 14.03 to 52.57. There was a significant effect of sampling time on the differences between measured masses and masses predicted with the minimal adequate GAM (*P* = 0.0034; Supplementary Material, Table S3 and Fig. 3). Differences did not differ between preoperative and any of the postoperative sampling times, but values of the 6 h postoperative sampling time differed from those of immediately postoperative sampling (*P* = 0.002) and 12 h postoperative sampling (*P* = 0.033). There was no fixed bias except at 6 h postoperative which amounted to less than 10% of the spread at this sampling time. The spread varied from 6.85 to 11.07.

## DISCUSSION

This study attempted to establish a routine to convert CK-MB activities into CK-MB masses for the evaluation of periprocedural myocardial damage in multicentre trials, using CK-MB levels on a common scale. Linear regression resulted in an acceptable agreement of predicted and measured masses, which varied by sampling time in terms of spread and fixed bias. A GAM with sex and sampling time as additional factors provided a superior agreement with a narrower spread between the LoAs, a marginal fixed bias at a single sampling time, and no proportional bias.

The knowledge about the extent and time course of cardiac biomarkers indicating myocardial cell damage following surgical interventions on the heart is of utmost importance for the successful perioperative treatment. The distinction between a reversible injury and definite myocardial infarction inevitably leading to scar and loss of function is crucial for the fate of the patients [4].

The mode of cardiac injury (global but temporary with myocardial protection in cardiac operations vs local but lasting in ischaemic myocardial infarction) necessitates a differentiated view on cardiac biomarkers. Procedural myocardial injury is observed regularly after cardiac operations. However, a specific cut-off value of a distinct biomarker differentiating a periprocedural myocardial infarction from “normal” injury is hard to define due to various types of surgery (coronary vs non-coronary) and different test assays.

To date the most sensitive biomarker in detecting myocardial injury in ACS is cardiac troponin I which is almost exclusively expressed in the heart [20]. Elevated cardiac troponin values reflect injury to myocardial cells irrespective of the pathophysiological mechanisms. A variety of pathophysiological mechanisms (e.g. myocardial shear stress, cardioplegia, oedema, inflammation, haemodilution, air embolism) are responsible for temporary rises in cardiac troponins even in otherwise normal hearts. Cardiac troponin levels could be elevated in patients with renal failure or chronic kidney disease [21].

The MB isoenzyme of creatine kinase is the most practical, robust and reliable biomarker for detecting perioperative myocardial injury. Unlike cardiac troponins, CK-MB is expressed also in the skeletal muscles (2–5% of all CK) but mostly in cardiomyocytes [1]. CK-MB is less sensitive to non-ischaemic causes of myocardial injury and independent of renal function. Currently, CK-MB is the preferred biomarker in clinical studies focusing on the quality of myocardial protection during cardiac operations using extracorporeal circulation.

Lévesque *et al.* [7] had established a linear relationship between CK-MB activity and mass data obtained with 2 commercial diagnostic CK-MB assays. Although the coefficient of determination was excellent and there was a negligible offset, the Bland–Altman plot showed a proportional bias which would underestimate higher CK-MB activities compared to the corresponding masses. The initial motivation for this study was to re-establish this relationship for 2 currently available diagnostic assays which will be used in different study centres of the SECRET multicentre trial.

The distribution of CK-MB activity and mass data was non-normal as expected. There was a dense cloud of readings, including all preoperative samples, at the low end of the scatter plot whereas only few patients showed high readings post-operatively (Fig. 1). Figure 3C indicates that the activity per mass was exceptionally high in samples with a very low CK-MB mass. This may stem from the lower sensitivity of the activity assay compared to the mass assay. This also explains the significant offset of 12.6 µg/l in the OLS regression model. Accordingly, the data of the present study showed a lower coefficient of determination between the activity and mass data compared to Lévesque *et al.* [7]. We hypothesized that the inclusion of these covariates in an appropriate non-parametric model might improve the prediction of masses from activities. GAMs describe a dependent variable as the sum of weighted smoothing functions calculated from the main predictor and covariates, optionally with terms that model interactions and address random effects such as those that arise from patient heterogeneity. As the reference ranges of CK-MB masses and activities differ between sexes [11, 19], the model was specified with smoothers stratified by sex. The influence of surgery, resulting in different readings per patient at the given sampling times, was modelled with an appropriate random error term. Age, BMI and all intraoperative factors were found to have no significant effect in the model. The minimal adequate model thus contained only the CK-MB activity as the main predictor, and sex and sampling time as covariates. This model provided an excellent goodness of fit to the empirical data with an even higher coefficient of determination compared to previous studies [7, 9]. The inclusion of sex as a covariate improved the fit considerably over an equivalent model without this covariate (not shown). The importance of the covariates sex and sampling time can be demonstrated by a comparison with the simple GAM. The replacement of the linear relationship in the OLS regression with a non-parametric GAM smoother did improve the fit as judged by the *r*^2^ values, but the fit was still far from that of the minimal adequate GAM. The Bland–Altman analysis of CK-MB mass data predicted with this model against the measured masses showed a spread between the limits of agreement which was almost 4-fold lower than in a linear regression-based prediction. There was no fixed or proportional bias. The former indicates that the prediction does not systematically over- or underestimate masses from all ranges of activities. The latter indicates that the agreement between predicted and measured CK-MB masses does not depend on the magnitude of the mass.

The validity of the present study may be hampered by several limitations. The limited number of planned patients was further decreased by the exclusion of two patients from the analysis. The entire dataset contained only few patients with extremely high CK-MB activities and masses. These patients show an above-average leverage on the predicted data. Lévesque *et al.* [7] included samples retrospectively and covered the entire expected range evenly. This study was conducted as a prospective trial instead and tried to address the expected lower frequency of medium and high CK-MB levels by using three postoperative sampling times per patient in addition to the usually low preoperative levels in elective patients. The data distribution can thus be expected to be representative for the SECRET trial data. The present model does not cover excessively high CK-MB levels which may occur during postoperative ACS because there were no ACS cases. We consider it mandatory to recalculate the model parameters, and potentially to repeat the factor selection, for patient cohorts differing from the one investigated in this study.

In summary, this study demonstrated the utility of GAMs for improving the conversion of clinical CK-MB data determined with different types of assays. This allows the use of CK-MB levels from different study centres on a common scale for statistical modelling purposes. This approach can be transferred to other data conversion problems. If the relationship of the underlying data does not permit a sufficient goodness of fit with linear models, non-parametric alternatives may provide a solution with a manageable additional computational effort.

## Supplementary Material

ivae138_Supplementary_Data

## Data Availability

Deidentified study data containing all parameters used in this manuscript are provided under the CC BY 4.0 International license in an open access repository [22]. **Markus Hoenicka:** Formal analysis; Resources; Writing—original draft. **Arbresha Vokshi:** Data curation; Investigation; Writing—review and editing. **Shaoxia Zhou:** Investigation; Resources; Writing—original draft. **Andreas Liebold:** Conceptualization; Project administration; Writing—review and editing. **Benjamin Mayer:** Conceptualization; Formal analysis; Writing—review and editing. Interactive CardioVascular and Thoracic Surgery thanks Tomislav Kopjar and the other anonymous reviewers for their contribution to the peer review process of this article.

## ABBREVIATIONS

ACS: Acute coronary syndrome
AIC: Akaike's information criterion
BMI: Body mass index
CK-MB: Creatine kinase MB
GAM: Generalized additive model
GCV: Generalized cross-validation
OLS: Ordinary least squares
SD: Standard deviation
