# Supplementary material for: Feasibility of aligning creatine kinase MB activity and mass data in multicentre trials using generalized additive modelling
Source: Interdiscip Cardiovasc Thorac Surg. 2024 Jul 23;39(2):ivae138. doi: 10.1093/icvts/ivae138 (PMC11298413; doi:10.1093/icvts/ivae138)
Supplement: ivae138_Supplementary_Data [file ivae138_supplementary_data.docx]

# Improved conversion of creatine kinase MB (CK-MB) activity into mass data by generalized additive modeling

- 1. **Supplemental data**

Markus Hoenicka, Arbresha Vokshi, Shaoxia Zhou, Andreas Liebold, Benjamin Mayer

## Models

### Model 1: Ordinary least squares linear regression model

Call:

lm(formula = ckmb$CK_MB_Act ~ ckmb$CK_MB_Mass)

Residuals:

Min 1Q Median 3Q Max

-12.730 -5.225 -2.869 2.408 79.506

Coefficients:

Estimate Std. Error t value Pr(>|t|)

(Intercept) 12.58607 1.05865 11.89 <2e-16 ***

ckmb$CK_MB_Mass 1.10040 0.03998 27.52 <2e-16 ***

---

Signif. codes: 0 ‘***’ 0.001 ‘**’ 0.01 ‘*’ 0.05 ‘.’ 0.1 ‘ ’ 1

Residual standard error: 9.792 on 150 degrees of freedom

Multiple R-squared: 0.8347, Adjusted R-squared: 0.8336

F-statistic: 757.6 on 1 and 150 DF, p-value: < 2.2e-16

### Model 2: Simple generalized additive model

Family: gaussian

Link function: identity

Formula:

CK_MB_Mass ~ s(CK_MB_Act)

Parametric coefficients:

Estimate Std. Error t value Pr(>|t|)

(Intercept) 17.5079 0.5886 29.74 <2e-16 ***

---

Signif. codes: 0 ‘***’ 0.001 ‘**’ 0.01 ‘*’ 0.05 ‘.’ 0.1 ‘ ’ 1

Approximate significance of smooth terms:

edf Ref.df F p-value

s(CK_MB_Act) 7.599 8.487 116.7 <2e-16 ***

---

Signif. codes: 0 ‘***’ 0.001 ‘**’ 0.01 ‘*’ 0.05 ‘.’ 0.1 ‘ ’ 1

R-sq.(adj) = 0.867 Deviance explained = 87.4%

GCV = 55.824 Scale est. = 52.665 n = 152

### Model 3: Maximal generalized additive model

Family: gaussian

Link function: identity

Formula:

CK_MB_Mass ~ s(CK_MB_Act, by = Sex) + s(Age_y, by = Sex) +

s(BMI, by = Sex) + s(Surgtime_min, by = Sex) +

s(Bypasstime_min, by = Sex) + s(CCtime_min, by = Sex) +

s(Samplingtime, k = 3) + ti(Samplingtime, CK_MB_Act, bs = "re") +

ti(Patient, CK_MB_Act, bs = "re")

Parametric coefficients:

Estimate Std. Error t value Pr(>|t|)

(Intercept) 15.053 1.363 11.04 <2e-16 ***

---

Signif. codes: 0 ‘***’ 0.001 ‘**’ 0.01 ‘*’ 0.05 ‘.’ 0.1 ‘ ’ 1

Approximate significance of smooth terms:

edf Ref.df F p-value

s(CK_MB_Act):Sexm 6.1883 7.166 16.329 <2e-16 ***

s(CK_MB_Act):Sexf 4.8740 5.701 26.704 <2e-16 ***

s(Age_y):Sexm 1.0000 1.000 0.756 0.3867

s(Age_y):Sexf 1.0000 1.000 0.624 0.4314

s(BMI):Sexm 1.0000 1.000 0.270 0.6046

s(BMI):Sexf 1.2694 1.394 0.162 0.6413

s(Surgtime_min):Sexm 1.0000 1.000 0.671 0.4147

s(Surgtime_min):Sexf 1.0000 1.000 0.123 0.7262

s(Bypasstime_min):Sexm 1.0000 1.000 0.078 0.7811

s(Bypasstime_min):Sexf 1.0000 1.000 1.450 0.2313

s(CCtime_min):Sexm 1.0000 1.000 0.001 0.9751

s(CCtime_min):Sexf 1.0000 1.000 1.792 0.1837

s(Samplingtime) 1.8335 1.965 5.517 0.0112 *

ti(Samplingtime,CK_MB_Act) 0.8477 1.000 4.016 0.0200 *

ti(Patient,CK_MB_Act) 27.3853 37.000 5.953 <2e-16 ***

---

Signif. codes: 0 ‘***’ 0.001 ‘**’ 0.01 ‘*’ 0.05 ‘.’ 0.1 ‘ ’ 1

R-sq.(adj) = 0.98 Deviance explained = 98.7%

GCV = 12.057 Scale est. = 7.9005 n = 152

### Model 4: Minimal adequate generalized additive model

Family: gaussian

Link function: identity

Formula:

CK_MB_Mass ~ s(CK_MB_Act, by = Sex) + s(Samplingtime, k = 3) +

ti(Samplingtime, CK_MB_Act, bs = "re") + ti(Patient, CK_MB_Act,

bs = "re")

Parametric coefficients:

Estimate Std. Error t value Pr(>|t|)

(Intercept) 13.532 1.438 9.412 1.36e-15 ***

---

Signif. codes: 0 ‘***’ 0.001 ‘**’ 0.01 ‘*’ 0.05 ‘.’ 0.1 ‘ ’ 1

Approximate significance of smooth terms:

edf Ref.df F p-value

s(CK_MB_Act):Sexm 8.689 8.925 13.952 < 2e-16 ***

s(CK_MB_Act):Sexf 5.053 5.913 29.598 < 2e-16 ***

s(Samplingtime) 1.837 1.969 4.316 0.01370 *

ti(Samplingtime,CK_MB_Act) 1.000 1.000 8.141 0.00272 **

ti(Patient,CK_MB_Act) 30.232 37.000 8.243 < 2e-16 ***

---

Signif. codes: 0 ‘***’ 0.001 ‘**’ 0.01 ‘*’ 0.05 ‘.’ 0.1 ‘ ’ 1

R-sq.(adj) = 0.981 Deviance explained = 98.7%

GCV = 10.816 Scale est. = 7.4137 n = 152

## Tables

1. Table 1: Demographic data of n=38 analyzed patients. Continuous numeric data are presented as median (1st quartile to 3rd quartile) for non-normally distributed data, and as mean (SD: standard deviation) for normally distributed data. Frequencies are presented as counts (percent of total).

| 1. **Parameter** | 1. **value** |
| --- | --- |
| 1. **demographic factors** | |
| 1. age (years) | 1. 71 (59 to 74) |
| 1. sex (male / female) | 1. 28 (74%) / 10 (26%) |
| 1. height (cm) | 1. 172.9 (SD: 8.7) |
| 1. weight (kg) | 1. 86.1 (SD: 17.6) |
| 1. body mass index (kg·m^-2^) | 1. 28.6 (SD: 4.8) |
| 1. **risk factors** | |
| 1. hypertension | 1. 35 (95%) |
| 1. hyperlipidemia | 1. 31 (84%) |
| 1. familial disposition | 1. 13 (35%) |
| 1. smoking | 1. 10 (27%) |
| 1. diabetes mellitus | 1. 10 (27%) |
| 1. obesity | 1. 9 (24%) |
| 1. **procedures** | |
| 1. aortic valve replacement | 1. 21 (55%) |
| 1. mitral valve repair or replacement | 1. 5 (13%) |
| 1. tricuspid valve repair | 1. 2 (5%) |
| 1. coronary artery bypass grafting | 1. 22 (58%) |
| 1. aortic surgery | 1. 10 (26%) |
| 1. radio frequency ablation | 1. 4 (11%) |
| 1. combined procedures | 1. 20 (53%) |
| 1. **intraoperative data** | |
| 1. surgery time (min) | 1. 235.7 (SD: 58.9) |
| 1. bypass time (min) | 1. 120.0 (98.3 to 154.5) |
| 1. cross-clamp time (min) | 1. 67.5 (57.0 to 90.3) |

Table 2: Influence of sex and sampling time on creatine kinase isoenzyme MB (CK-MB) activity and mass data as well as on the ratio of both measurements. Data are presented as median (1st quartile to 3rd quartile). n=38 patients.

| **sampling time** | **sex** | **CK-MB activity (U/L)** | **CK-MB mass (µg/L)** | **ratio (U/µg)** |
| --- | --- | --- | --- | --- |
| preoperative | all  - male  - female | 10.02 (8.58 to 13.80)  11.10 (8.98 to 14.2)  8.78 (8.35 to 9.31) | 2.15 (1.50 to 2.94)  2.27 (1.59 to 3.22)  1.57 (1.29 to 2.30) | 4.91 (3.57 to 5.99)  4.36 (3.54 to 5.95)  5.49 (4.02 to 6.03) |
| postoperative | all  - male  - female | 38.70 (28.33 to 45.88)  35.45 (27.25 to 44.75)  41.50 (36.40 to 51.33) | 16.50 (11.33 to 21.38)  13.60 (10.53 to 20.10)  18.75 (14.63 to 28.65) | 2.13 (1.91 to 2.71)  2.15 (1.87 to 2.73)  1.97 (1.93 to 2.59) |
| 6 h postoperative | all  - male  - female | 27.90 (23.05 to 39.30)  26.80 (22.90 to 33.60)  31.45 (25.23 to 42.20) | 17.05 (10.73 to 24.10)  17.00 (10.45 to 22.35)  19.00 (12.68 to 24.10) | 1.64 (1.46 to 1.96)  1.60 (1.41 to 1.92)  1.86 (1.51 to 1.98) |
| 12 h postoperative | all  - male  - female | 27.50 (22.60 to 43.88)  26.60 (20.73 to 41.03)  30.70 (24.70 to 56.00) | 14.50 (11.10 to 21.55)  13.60 (10.46 to 24.18)  16.95 (14.15 to 21.45) | 1.74 (1.48 to 1.98)  1.80 (1.46 to 1.98)  1.67 (1.59 to 1.97) |

Table 3: Comparison of creatine kinase isoenzyme MB masses predicted from activity data with measured masses according to the Bland-Altman method. Masses were predicted with a ordinary least squares linear regression model (OLS) and with the minimal adequate generalized additive model (GAM) with sex and sampling time as additional factors. Comparisons were calculated for all data (152 samples from 38 patients at 4 sampling times) and for all 4 sampling times separately. ULoA, upper limit of agreement, LLoA, lower limit of agreement, CI, confidence interval. n=38 patients.

| **model** | **sampling time** | **samples (n)** | **ULoA (95% CI)**  **(µg/L)** | **LLoA (95% CI)**  **(µg/L)** | **Spread**  **(µg/L)** | **Fixed bias (95% CI)**  **(µg/L)** |
| --- | --- | --- | --- | --- | --- | --- |
| OLS | all times | 152 | 17.38 (14.95 to 19.82) | -17.38 (-19.82 to -14.95) | 34.77 | 0 (-1.42 to 1.42) |
|  | preoperative | 38 | 3.42 (1.39 to 5.45) | -10.61 (-12.64 to -8.58) | 14.03 | -3.59 (-4.77 to -2.42) |
|  | postoperative | 38 | 20.37 (16.02 to 24.72) | -9.70 (-14.05 to -5.36) | 30.07 | 5.33 (2.81 to 7.85) |
|  | 6 h postoperative | 38 | 7.78 (5.02 to 10.55) | -11.34 (-14.11 to -8.58) | 19.12 | -1.78 (-3.38 to -0.18) |
|  | 12 h postoperative | 38 | 26.33 (18.73 to 33.93) | -26.24 (-33.84 to -18.64) | 52.57 | 0.04 (-4.37 to 4.45) |
| GAM | all times | 152 | 4.43 (3.81 to 5.05) | -4.43 (-5.05 to -3.81) | 8.87 | 0 (-0.36 to 0.36) |
|  | preoperative | 38 | 3.30 (2.31 to 4.30) | -3.55 (-4.54 to -2.56) | 6.85 | -0.12 (-0.70 to 0.45) |
|  | postoperative | 38 | 5.03 (3.72 to 6.33) | -4.00 (-5.31 to -2.70) | 9.03 | 0.51 (-0.25 to 1.27) |
|  | 6 h postoperative | 38 | 3.14 (2.04 to 4.24) | -4.50 (-5.55 to -3.35) | 7.59 | -0.65 (-1.29 to -0.02) |
|  | 12 h postoperative | 38 | 5.80 (4.20 to 7.40) | -5.27 (-6.87 to -3.67) | 11.07 | 0.27 (-0.66 to 1.19) |
